# A Neuroscience Levels of Explanation Approach to the Mind and the Brain

**DOI:** 10.3389/fncom.2021.649679

**Published:** 2021-04-07

**Authors:** Edmund T. Rolls

**Affiliations:** ^1^Oxford Centre for Computational Neuroscience, Oxford, United Kingdom; ^2^Department of Computer Science, University of Warwick, Coventry, United Kingdom

**Keywords:** psychiatry, the mind-brain problem, causality, neuronal networks, neural computation, philosophy of mind, computational neuroscience of mind, consciousness

## Abstract

The relation between mental states and brain states is important in computational neuroscience, and in psychiatry in which interventions with medication are made on brain states to alter mental states. The relation between the brain and the mind has puzzled philosophers for centuries. Here a neuroscience approach is proposed in which events at the sub-neuronal, neuronal, and neuronal network levels take place simultaneously to perform a computation that can be described at a high level as a mental state, with content about the world. It is argued that as the processes at the different levels of explanation take place at the same time, they are linked by a non-causal supervenient relationship: causality can best be described in brains as operating within but not between levels. This allows the supervenient (e.g., mental) properties to be emergent, though once understood at the mechanistic levels they may seem less emergent, and expected. This mind-brain theory allows mental events to be different in kind from the mechanistic events that underlie them; but does not lead one to argue that mental events cause brain events, or vice versa: they are different levels of explanation of the operation of the computational system. This approach may provide a way of thinking about brains and minds that is different from dualism and from reductive physicalism, and which is rooted in the computational processes that are fundamental to understanding brain and mental events, and that mean that the mental and mechanistic levels are linked by the computational process being performed. Explanations at the different levels of operation may be useful in different ways. For example, if we wish to understand how arithmetic is performed in the brain, description at the mental level of the algorithm being computed will be useful. But if the brain operates to result in mental disorders, then understanding the mechanism at the neural processing level may be more useful, in for example, the treatment of psychiatric disorders.

## Introduction

The relation between events at different levels, such as the sub-neuronal, neuronal, neuronal network, and even mental levels is important for computational neuroscience. How does causality operate in such a system? What is the relation between brain events and mental events?

Philosophers have long been thinking about the relation between the mind and the brain. Descartes took a dualist approach (Descartes, 1644), and that raised the problem of how the mind and brain relate to each other.

Searle (1992) had a clear opinion: “It seems to me obvious from everything we know about the brain that macro mental phenomena are all caused by lower-level micro phenomena. There is nothing mysterious about such bottom-up causation: it is quite common in the physical world. Furthermore, the fact that mental features are supervenient on neuronal features in no way diminishes their causal efficacy. The solidity of a piston is causally supervenient on its molecular structure, but this does not make solidity epiphenomenal; and similarly, the causal supervenient of my present back pain on micro events in my brain does not make the pain epiphenomenal. My conclusion is that once you recognise the existence of bottom-up, micro to macro forms of causation, the notion of supervenient no longer does any work in philosophy.” (Supervenience literally means “coming on top of,” and specifies conditions that should hold between the higher level and lower level properties. Supervenience in our case specifies that a set of mental properties M1 supervenes upon another (physical) set P1 just in case no two things can differ with respect to M-properties without also differing with respect to their P-properties (McLaughlin and Bennett, 2011)).

Starting with an interventionist account of causation (Woodward, 2005), Baumgartner (2009) in a “causal exclusion” argument held that the assumption of a supervenience relation violates the criterion of what counts as a good intervention. He held that as a result, we cannot draw conclusions about the causal relation between mental states and brain states. In his reply to Baumgartner (2009), Woodward (2015) proposed to adjust these intervention criteria in order to make room for supervenience relations and to secure causal claims for cognitive neuroscience investigations (see Dijkstra and De Bruin, 2016). (The interventionist theory of causality is roughly that *C* causes *E* if and only if an intervention on *C* would bring about a change in *E*. An intervention on *C* means some manipulation of *C* that changes it. *C* is a variable that can take different states).

The relation between mental events and brain events (Kim, 1998, 2011) is also important in psychiatry (Dijkstra and De Bruin, 2016). For example, we may find that functional connectivities between some brain areas are different in schizophrenia and depression, and are correlated with the symptoms (Rolls et al., 2020; Rolls, 2021a). Moreover, in some brain areas, the neural (fMRI BOLD signal) activations to a set of stimuli are directly correlated with their subjective pleasantness (Grabenhorst and Rolls, 2011; Rolls, 2018a, 2019, 2021b), and these reward systems appear to operate abnormally in depression (Rolls et al., 2020). Another example of the relation between the brain and the mind is that damage to the orbitofrontal cortex impairs emotional behavior (Bechara et al., 2000; Fellows, 2011; Rolls, 2019; Rolls et al., 2020) and subjective emotional experience (Hornak et al., 2003). In these examples, does the difference in the brain cause the difference in the mental including subjective states? This is important for treatment, for it may be useful to know whether a possible treatment might be to reduce the differences in the brain, using for example, medication (Rolls, 2021a), and this raises the issue of how the mental states are changed. Psychiatry is one area in which developing our understanding of the relation between brain states and events, and mental states and events, may be useful (Dijkstra and De Bruin, 2016).

In this paper I develop and explain further a theory of the relation between events investigated at different levels of neuroscience (Rolls, 2020). These include ion channels in neurons activated by transmitters; the action potentials produced by neurons to transmit information to other neurons; and the collective activity of large populations of neurons to perform mental processes such as short-term memory, long-term memory, decision-making, and emotion. The ways in which these computations are performed are described elsewhere (Rolls, 2021b). Here I focus on the relations between the activity at different levels of these processes. I argue that these are different levels of explanation, each with their uses. As a neuroscientist, I argue that we can think of causality as operating within a level, but not between levels. That is an important move, and if accepted helps enormously with the great debates about dualism, functionalism, physical reductionism, etc. In this paper I reach as far as mental events, but do not deal with phenomenal consciousness, which I have considered elsewhere (Rolls, 2004, 2012b, 2020). This is clarified below, but a mental event as considered here does not include what is experienced phenomenally (Rolls, 2020); but the mental events considered here can include events such as perception, memory, and emotional states that, as shown below, can have content.

## Levels of Explanation in Neuroscience, and the Relation Between the Mind and the Brain

We can now understand brain processing from the level of ion channels in neurons, through neuronal biophysics, to neuronal firing, through the computations performed by populations of neurons, and how their activity is reflected by functional neuroimaging, to behavioral and cognitive effects (Rolls, 2016b, 2021b). Activity at any one level can be used to understand activity at the next. This raises the philosophical issue of how we should consider causality with these different levels (Rolls, 2016b, 2020, 2021b). Does the brain cause effects in the mind, and do events at the mental, mind, level cause brain activity?

What is the relation between the mind and the brain? This is the mind-brain or mind-body problem. Do mental, mind, events cause brain events? Do brain events cause mental effects? What can we learn from the relation between software and hardware in a computer about mind-brain interactions and how causality operates?

Some have argued that phenomenal consciousness can be reduced to a natural (physical or physically realized) property (Carruthers, 2019). This, for him, takes much of the mystery out of phenomenal consciousness, for it is just a matter of matter, and simplifies his approach to all the questions raised by phenomenal consciousness. But is it reasonable to argue that one can reduce what is at a very high level in the processing system to the physical properties that implement the processing at a lower level? I do not think so. To make this point, we need to consider how different levels of the system, such as the neuronal level and the computational function being performed, relate to each other. This is part of the very big problem of the relation between the mind and the brain. Here is my approach to this.

One possible view that has been described (Rolls, 2016b) is that the relationship between mental events and neurophysiological events is similar to the relationship between the program running in a computer and the hardware of the computer. Does the program (the software loaded onto the computer usually written in a high-level language such as C and then compiled into machine code “cause” the logic gates of the computer hardware to move to the next state? And does this hardware state change “cause” the program to move to its next step or state? It would be helpful if those interested in the philosophy of the “mind-brain” problem would provide, as a starting point, a clear view on this computational issue in computers, as that is a well-formulated problem. Once there is satisfactory explanation here, one is likely to be in a better position to understand the mind-brain problem.

I propose that one way to think about this is that when we are looking at different levels of what is overall the operation of a computational system, causality can usefully be understood as operating within levels (causing one step of the program to move to the next; or the neurons to move from one state to another), but not between levels (e.g., software to hardware and vice versa). That is, if the events at the different levels of explanation are occurring simultaneously, without a time delay, then my view is that we should not think of causality as operating between levels, but just that what happens at a higher level may be an emergent property of what happens at a lower level. This is the solution I propose to this aspect of the mind-brain problem.

Following this thinking, when one step of a process at one level of explanation moves to the next step in time, we can speak of causality that would meet the criteria for Granger causality where one time series, including the time series being considered, can be used to predict the next step in time (Granger, 1969; Bressler and Seth, 2011; Ge et al., 2012). In contrast, when we consider the relationship between processes described at different levels of explanation, such as the relation between a step in the hardware in a computer and a step in the software, then these processes may occur simultaneously, and be inextricably linked with each other, and just be different ways of describing the same process, so that temporal (Granger) causality does not apply to this relation between levels, but only within levels. The whole processing can now be specified from the mechanistic level of neuronal firings, etc., up through the computational level to the cognitive and behavioral level.

Let me develop this further. In neuroscience (and this may be different from quantum physics), we think that when causes produce effects a time delay is a useful indicator. For example, if I present a face to an individual, neurons in the primate primary visual cortex V1 may start firing within 30–40 ms, in V2 approximately 15 ms later, in the next stage V4 15 ms later, in posterior inferior temporal cortex 15 ms later, and in the anterior inferior temporal after another 15 ms at approximately 90–100 ms after the stimulus is shown (Rolls, 1992, 2021b). I interpret this, knowing too the anatomical connections in this serial hierarchy, and the effects of damage to each of the stages, that the firing in V1 causes the firing in V2 etc., all the way up to the anterior inferior temporal cortex, where face selective neurons are found (Perrett et al., 1982; Rolls, 1984). We can say that a representation of individual faces that is invariant with respect to size, position and even view has been formed by the inferior temporal visual cortex (Rolls, 2012a, 2021b). This is a representation of a face in the world as shown by the mutual information between the neuronal firing and the face (Rolls and Treves, 2011; Rolls, 2021b). We can say that this representation has content (Shea, 2018), because of the effects that are produced by damage to this region or to regions to which it projects. For example, damage to the human fusiform face cortex produces an inability to recognize faces (prosopagnosia); and damage to the orbitofrontal cortex which receives inputs from the inferior temporal cortex produces impairments in face expression identification (Hornak et al., 1996, 2003). So mental events are involved, in this case related to perception and emotion. However, what happens within an area such as the inferior temporal cortex where the invariant face representation is formed is that the synaptic inputs are received from the posterior inferior temporal visual cortex, ion channels in the neurons are opened, the neurons start to fire action potentials, and then there is a competition between the excitatory neurons (pyramidal cells) implemented by feedback inhibition from the inhibitory neurons until a sparse distributed representation of relatively few neurons firing at relatively high rates is produced (Rolls, 2021b). This categorization is even helped by recurrent excitatory connections between the excitatory neurons that help the population acting as an attractor network to fall into a representation of one or a different person or object learned in previous experience (Akrami et al., 2009). This collective computation performed by populations of neurons can all take place within 15–20 ms in a cortical area (Panzeri et al., 2001; Rolls, 2016b, 2021b). The argument is that a computation has been performed that can be described at the synaptic, neuronal, and neuron population level, and all happen at the same time and are accompanied at the same time by what can be described as the mental process of invariant object recognition, which, as we have seen, carries content.

Another example of the type of mental state I refer to here is recall of a whole memory. The theory is that the CA3 neurons of the hippocampus with their recurrent excitatory associatively modifiable synaptic connections implement an attractor network used in the retrieval of the whole of an episodic memory from any part of it (Rolls, 1989, 2018b, 2021b; Kesner and Rolls, 2015). The retrieval cue, a small fraction of the original memory, for example, the place, when the whole episodic memory is about seeing a person in a particular place, is applied via the perforant path to the CA3 neurons. This recall cue consists of a subset of the original perforant path neurons firing. This firing releases glutamate as a transmitter that opens ion channels in the CA3 neurons for which these synapses are strong. The opening of the ion channels depolarizes the membrane potentials of these CA3 neurons, and they start to fire action potentials. These action potentials then activate other CA3 neurons with strong synapses that were strengthened because they were coactive during the learning of the episodic memory originally. So the full pattern of activity of the CA3 neurons, which will now include the person who was present at that place originally, starts to be recalled (Rolls, 2021b). The recall then is a collective computation in which all the CA3 neurons with strengthened synapses between them learned during the original episode fall into the optimal basin of attraction (with inhibitory feedback neurons preventing this excitatory interaction of the CA3 cells from blowing up with all the neurons active). This collective computation occurs across the whole population of neurons in what is termed an attractor network, and this will find the optimal recall as shown with the techniques of theoretical physics (Hopfield, 1982; Amit, 1989; Treves and Rolls, 1994; Rolls, 2021b). This retrieval of a full memory in the hippocampus, and its recall even to the neocortical areas that originally had neuronal activity during the learning of the episodic event (Rolls, 1989, 2018b, 2021b; Treves and Rolls, 1994), is a mental operation, that of the recall of a whole episodic memory from any part of it. Moreover, this has mental content (Shea, 2018), in that interference with this hippocampal process impairs memory (Kesner and Rolls, 2015; Feng et al., 2020). So we have seen in this example how different levels of explanation, from ion channels, through neuronal firing, though the collective computation performed by the whole population of 300,000 CA3 neurons, can result in the correct recall of any one of approximately 10,000 memories [values for the rat (Treves and Rolls, 1994; Rolls, 2021b)]. The so-called “emergent” events of full memory recall at the level of the whole population of neurons (Hopfield, 1982), which is a mental or mind-level event, can best be understood at the level of explanation of the collective properties of the whole neuronal population operating in the way described; but supervene on the lower levels such as the firing of individual neurons. The neurons step in time from one subset firing to another until the full set is complete, with causality at this level; and the mental state moves from an incomplete recall cue to a full memory being present, with again causality operating at this level. The collective computation is related to the firing of the neurons, and is different in **kind** to the firing of the neurons. So what occurs at the different levels of explanation can be described as supervenience of one level on another, with causality operating across time within a level but not between levels, because the mental and neuronal processes occur at the same time.

Timing and its relation to causality do seem relevant to this discussion, so it may be helpful to consider what happens in a computer in a little more detail when a program runs from one step to another in the computer. At the program level, we can assume that one step of the compiled program (e.g., add *a* to *b*, i.e., *b* = *b* + *a*) causes *a* to be added to *b*. The compiled code is read by automaton-like operations performed by the Control Unit (CU) such as copy the contents of memory from where *a* is stored to a register in the central arithmetic logic unit (ALU), copy the contents of memory from where *b* is stored to a register in the ALU, perform a logical operation such as ADD with the operands now in the registers of the ALU, and then with the CU copy the contents of the ALU back to the memory location where *b* is stored. We can say at the machine level that a series of steps is performed by the CU with each step following each other in a causal chain. So causality operates within that level. And at the next level down, within the ALU, logic gates work together to perform operations such as AND, OR, NOT, and XOR to then implement the mathematical functions such as ADD, MULTIPLY, SUBTRACT, and DIVIDE. In this system, the different levels all happen at the same time: *a* + *b* is calculated at the program level; *a* and *b* are fetched at the machine instruction level; and many operations are performed at the level of logic gates. But I argue that all these levels happen at the same time, not in a causal chain up and down between levels, involving time delays. The program level does not perform a step and then wait some time for a causal effect to occur in the CU. Instead, the CU performs its processes of reading the next step from the compiled program, fetching contents from memory addresses, letting the ALU operate, and then sending back the result to a memory address, all of which implements one step at the program level. So I do not wish to argue for causality between levels, as the timing does not support that. Thus my view is that we have a system that operates at different levels of explanation, with causality, and time, moving forward within each level, but without causality and time delays between levels. What happens at the different levels is however locked together, so that one level can be said in a non-causal sense to be supervenient on the level below.

Of course I accept that causality operates within a level. So a programmer decides that an algorithm requires *a* to be added to *b*. The programmer then uses a compiler such as C to include this in a program. The programmer then starts the compiled program to run by touching a key on the keyboard or moving the mouse and clicking. So all of these steps are causal, and operate within a given level of explanation, and serially in time.

Now we can explain what happens in this programming situation at any level of explanation that we choose: at the level of the programmer; or of the operation of stepping through the machine code by the computer CU; or at the level of the logic gate states that occur in the ALU.

When we describe the operation of the system, it may be very convenient to describe it at a particular level of explanation (Dennett, 1991). For example, the easiest way to understand what is happening in this computer example in terms of causality is to take the program level, “add *a* to *b*” (and not for example at the logic gate level). But to return to brains: it does matter greatly that we understand causally what is happening within each level of operation. For example, if the events at the subjective, mental, level operate with less stability so that cognitive symptoms arise in schizophrenia, then it can be very important to know what is happening at the level of brain connectivity and its implications for the stability of attractor networks, for this may provide not only an account for the mental symptoms, but also a way to treat them (Rolls, 2021a; Rolls et al., 2021).

**FIGURE 1:**
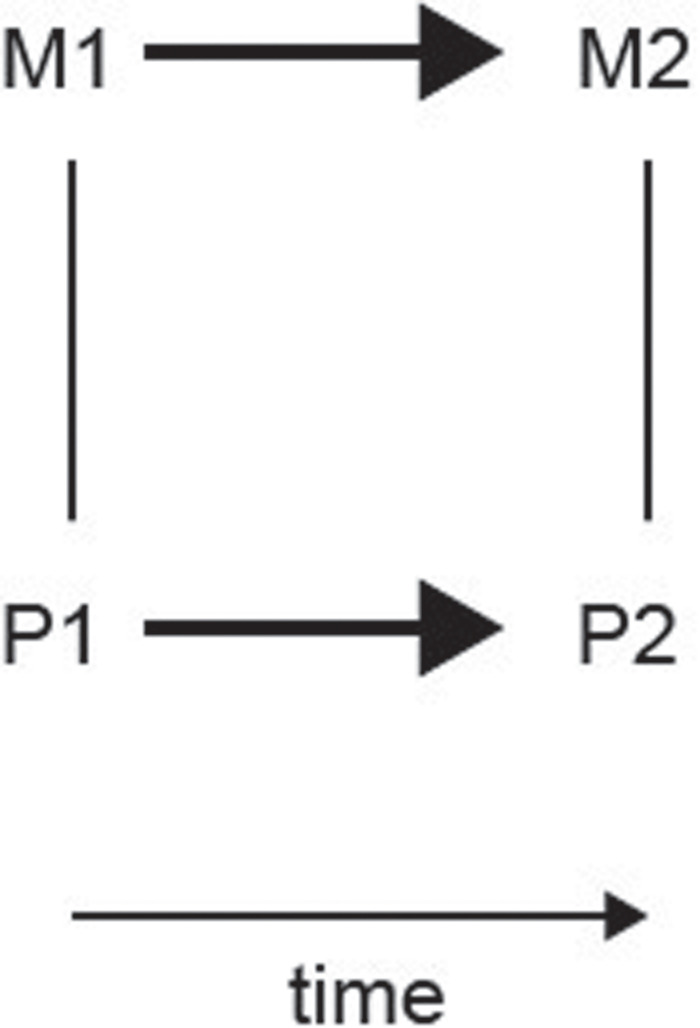
Schematic representation of the relation between physical brain states (P1 and P2) and mental states (M1 and M2). Undirected edges indicate supervenience/subvenience relations which apply upward and downward and are non-causal. The edges with an arrow indicate a causal relation.

The view I propose therefore is that different levels of explanation in at least computers and brains can be related to each other in a necessary (unique and obligatory) way (sometimes called supervenience), but that this relationship need not be causal, with a key argument that there is no time delay between the different levels of operation and explanation. There are approaches in philosophy that specify that mechanistic explanations may help to link levels of a system non-causally but “constitutively” (Craver and Bechtel, 2007; Craver and Tabery, 2015), but there has been discussion about what constitutes levels in those systems being considered (Eronen, 2013). There are also approaches in philosophy that aim to specify the conditions under which a physical system performs a computation defined by a mathematical formalism (Ritchie and Piccinini, 2018). Mappings between the physical and algorithmic levels are considered in this “computational implementation” approach, but the causal properties involved in these “mappings” were not detailed (Ritchie and Piccinini, 2018). What I have aimed to do here is to argue that explanations at each level of the system can be helpful in understanding the system, with causes even being considered to operate simultaneously at the different levels (Figure 1); and I have defined the levels at least for neuroscience at what seem to be clearly separate levels of operation, such as ion channels in neurons; the firing of neurons which transmits information to other neurons; the collective operation of large populations of neurons to fall into, for example, a stable coalition interpreted as an energy minimum that implements memory retrieval; and the description at a higher mental level of the fact that the memory has been recalled and can be reported (Rolls, 2021b).

Sometimes the cognitive effects that arise from brain computations (Rolls, 2021b) seem remarkable, for example, the recall of a whole memory from a part of it, and we describe this as an “**emergent property**,” but once understood from the mechanistic level upward, the functions implemented are elegant and wonderful, but understandable and not magical or poorly understood (Rolls, 2016b, 2021b). We can say here that the way in which a discrete attractor network settles by its collective computation into a low energy basin of attraction to solve a computational problem is different *in kind* from a set of individual neurons firing action potentials, or from the transmitter being released onto each of the 10,000 synapses in the typically 100,000 neurons in the cortical attractor network (Rolls, 2016b, 2021b). In this sense, I do not think that a claim that all the properties of the system, including its emergent properties, can be reduced to what might be happening in noisy ion channels in synapses. Of course, what is happening simultaneously at different levels of the system is essential for its operation. But what is happening at each level may be thought of as a different **kind**.

It may be helpful to place my thinking as a computational neuroscientist in a broader context. First, I argue here that cause and effect operate within a level of explanation, and not between the levels. That sidesteps problems of how the mind affects the brain (and vice versa), which involves issues of causality, and which has puzzled philosophers since Descartes (Bennett, 2007; Kim, 2011). It also leads to a position on the exclusion principle, which is that no effect has more than one sufficient cause unless it is overdetermined (Bennett, 2007; Kim, 2011). This principle has led to problems about mind-brain relations, for the question arises about whether mental events might be caused by mental events, and by brain activity, a physical event (Bennett, 2007; Kim, 2011). I argue that causality can be thought of as operating within each level of operation, and that causality in terms of computational neuroscience needs to be understood as operating within each of many different levels of explanation. Further, the presence of multiple routes to action which I have described in detail elsewhere (Rolls, 2014, 2020, 2021b) provides evidence that we need to know exactly which route to action was involved for a particular behavior: it is not sufficient to describe behavior as having inputs and outputs (Kim, 2011). The computations in between the input and output can be multiple, and even occurring at the same time, in parallel, in the brain (Rolls, 2021b). Second, I argue that different mental events are completely distinguishable by different neuronal events. The neuron-level events that use a sparse distributed representation across neurons are capable of encoding a large amount of information relevant to mental states (Rolls and Treves, 2011; Rolls, 2021b). Third, the brain is not just a syntactic processor: its neurons which are involved in brain computations convey by their firing rate specific content about the world, as shown by information theoretic analyses, and by the evidence that particular behavioral functions are impaired by damage to particular parts of the brain (Rolls and Treves, 2011; Rolls, 2021b). Indeed, each brain region provides a representation of different content (Rolls, 2021b). For example, some neurons in the orbitofrontal cortex respond to pleasant touch on the forearm produced by a slow gentle rub that is conveyed by C-tactile fibers (Rolls et al., 2003; McCabe et al., 2008; Rolls, 2010, 2016a; Olausson et al., 2016). Another set of C afferents respond to painful stimuli, and activates other parts of the orbitofrontal cortex (Rolls et al., 2003; Rolls, 2021b). The orbitofrontal cortex is causally involved in the emotion-related or affective sensations, which are impaired by damage to the orbitofrontal cortex (Hornak et al., 2003; Rolls, 2019). This point about content is an argument against Searle’s Chinese Room simile (Searle, 1982, 1990). Fourth, in assessing what mental states are possible, and are occurring, in a particular individual, we need to know what all the computational modules are that are involved, and how they operate, and what content is represented by their neuronal firing, to know whether the mental states are the same. The Turing test (Kim, 2011) is too behaviorist, as it is difficult to adequately ask questions that will assess all of the possible computational modules in the brain that are active together with their content when a particular behavioral output is produced.

The solution for me to know whether mental states are similar in different individuals is to know from neuroscience what the nature of each of the computational modules is in the relevant brain, and what their content is during a particular mental operation (Rolls, 2021b). The Turing test in principle may be useful, but in practice is not an easy way to know what mental events are taking place during a particular type of behavior in the individual. Instead, knowledge of the detailed computational architecture of the individual [e.g. whether the individual is capable of higher order syntactic thoughts (HOSTs), and what their contents are (Rolls, 2020)] is necessary in order to evaluate the mental states of different individuals, and how similar or not they may be. The brain/mental computational approach is thus the only approach that I think will suffice to elucidate questions such as whether two minds are similar or not, and whether the mental states that are involved, including phenomenal consciousness (Rolls, 2020), are similar or not. We need to know what computations are being performed by different brain regions; and then we will have a sound foundation for understanding mental states and how they require underlying brain states in particular computational modules with particular contents (Rolls, 2020, 2021b).

## Summary and Discussion

A summary of my theory of the relation between the mind and the brain is shown in Figure 1, for comparison with different views (Baumgartner, 2009; Woodward, 2015; Dijkstra and De Bruin, 2016). Physical brain states P1 and P2 are causally related such that P1 causes P2 at a later time. This is indicated by a directed arrow in Figure 1. Mental state M1 is supervenient on P1 in a non-causal relationship. A property of this supervenience is that a set of properties M1 supervenes upon another set P1 just in case no two things can differ with respect to M-properties without also differing with respect to their P-properties (McLaughlin and Bennett, 2011). Another property of my supervenience is that there is no time delay between P1 and M1, consistent with no causal relationship. Another property is that we can say that P1 is sub-convenient on M1: the relation applies both upward and downward between my different levels of explanation P and M. Similarly, mental state M2 is supervenient on P2 in a non-causal relationship. My suggestion is that causality can also be considered to operate between M1 and M2, again across time. My suggestion further is that because causality does not operate between different levels of explanation, it does not make sense to talk about M1 causing P2, or, for that matter, P1 causing M2. This is different to earlier views (Baumgartner, 2009; Kim, 2011; Woodward, 2015; Dijkstra and De Bruin, 2016).

What are the implications for dualist approaches to the mind and the brain? I argue that mental events are different in kind to their physical implementation. In a somewhat similar way, the program implemented in a computer, for example computing the square of a number, is different in kind to the currents flowing into the logic gates in a computer central processing unit. But I do not propose that mental events cause physical events, or vice versa: in my thinking, the causality operates within but not between levels. That avoids all the issues that arise if one tries to argue in a dualist system about how mental events cause physical events. In relation to the hard problem of phenomenal consciousness, I argue that this arises when a particular type of computation is being performed, HOSTs which can be used to correct first order syntactic thoughts (Rolls, 2004, 2012b, 2020).

The implications for psychiatry of the approach described here appear to be as follows. A treatment with medication of brain states can be considered as a cause (the medication) having effects on brain states by altering, for example, the effects of neurotransmitters. We can then say that at the same time as the brain states change, there are supervenient changes in mental states. The implication of the supervenience is that it is non-causal, but that the mental states are happening at a different level of explanation, with the mental states so closely linked to the brain states that a change in the brain states will be associated with changes in the mental states. This approach helps reconciliation of the concepts that mental states are different in kind from brain states; that mental states happen at the same time as brain states; and that the nature of the link is that mental and brain states are different levels of explanation.

## Data Availability Statement

The original contributions presented in the study are included in the article, further inquiries can be directed to the corresponding author.

## Author Contributions

The author confirms being the sole contributor of this work and has approved it for publication.

## Conflict of Interest

The author declares that the research was conducted in the absence of any commercial or financial relationships that could be construed as a potential conflict of interest.
